# Multiparametric magnetic resonance in the assessment of the gender differences in a high-grade glioma rat model

**DOI:** 10.1186/s13550-014-0044-4

**Published:** 2014-09-09

**Authors:** Rocío Pérez-Carro, Omar Cauli, Pilar López-Larrubia

**Affiliations:** Laboratory of Magnetic Resonance in the Study of the Central Nervous System, Instituto de Investigaciones Biomédicas ‘Alberto Sols’, CSIC-UAM, Arturo Duperier 4, 28029 Madrid, Spain; Department of Nursing, University of Valencia, 46010 Valencia, Spain

**Keywords:** Magnetic resonance imaging, Magnetic resonance spectroscopy, High-grade glioma, Blood-brain barrier, Gender-dependent markers

## Abstract

**Background:**

Glioblastoma, the most frequent and aggressive of all astrocytomas, presents a clear predominance in male humans, but the assessment of sexual differences in its tumourigenesis and growth has received little attention so far. In this study, we aim to identify gender-dependent surrogate markers in an animal model of this cancer by means of magnetic resonance (MR) imaging and biochemical and behavioural studies.

**Methods:**

A high-grade glioma model developed in male and female rats was used. Multiparametric magnetic resonance images and localized spectra were acquired. The MR parameters linked to tumoural features were quantified. Motor and metabolic activity was also assessed. Postmortem analyses were carried out to measure indicators of malignancy, tumoural metabolism and viability of the blood-brain barrier (BBB).

**Results:**

Statistically significant differences dependent on the animal sex were found in the study of pathological indicators like oedema, inflammation, cellularity and microvasculature. Results suggest higher cell proliferative rate, inflammation and vasogenic oedema and or necrosis in glioma-bearing male rats. Haemodynamic parameters measured indicated a major disruption of the BBB, postmortem confirmed, in this sex. Metabolomic and energetic metabolism activity data are in agreement with a major malignancy and aggressiveness of this cancer model on males.

**Conclusions:**

Gender differences should be taken into account in preclinical studies of glioblastoma models, in the characterization of the tumoural behaviour and consequently in the development and validation of new therapeutic approaches. MR imaging and spectroscopy allow to non-invasively monitor this sexual dimorphism in the diagnosis and prognosis of brain cancer.

## Background

Despite decades of intensive research, high-grade gliomas (WHO grade III and IV) [1] are currently considered incurable with a poor or very poor survival. The mean survival for these tumours remains, on average, 1 year for glioblastoma patients and from 2 to 3 years for anaplastic astrocytoma after diagnosis [2]. Glioblastoma multiforme (GBM) is the most frequent, aggressive and lethal intracranial tumour, accounting for approximately 50% of all astrocytomas [3]. This glioma is defined by a marked genetic instability, complete morphological and metabolic reprogramming, fast and uncontrolled proliferation, intense resistance to apoptosis, diffuse infiltration, robust angiogenesis and propensity to necrosis. Despite advances in multimodal therapeutic options [4], the median survival after diagnosis of GBM is only 14.6 months [5].

Despite the clear predominance of GBM tumours in the male population, the impact of these differences in brain tumourigenesis has received little attention to date. Particularly, there is a lack of gender-based studies with regard to brain tumour evolution and response to treatment. McKinley et al. reported in 2000 that males are 1.5 to 2 times more likely to develop glioblastoma compared with females [6]. Later, based on epidemiological data from 15 different countries on 6 continents, Sun et al. described a clear predominance of brain tumours in males compared to females with a ratio ranging from >1 to 3.5 [7]. They indicated that sex affects brain tumourigenesis through mechanisms that are likely to be independent of race and regional differences in environmental exposures. The same study established that the influence of gender on brain tumourigenesis is operative regardless/independent of age and sexual maturity, suggesting that it is not a consequence of the acute effects of circulating sex hormones. Interestingly, a positive association between late menarche and increased risk of glioma has been reported [8,9]. Furthermore, experimental studies show that oestrogens and their modulators inhibit proliferation of gliomas *in vivo* and induce cell death [10-13]. All these works suggest that genetic differences between males and females may play a role in the pathogenesis of this tumour. Therefore, the molecular mechanisms responsible for the development of gliomas in the different genders should be further evaluated. These studies could have a significant impact on our understanding of brain tumourigenesis and on therapeutic stratification.

Nowadays, magnetic resonance imaging (MRI) plays a key role in the detection, staging, characterization of the tumour infiltration and therapy validation of gliomas [14-16]. MRI techniques are commonly used to measure brain tumour growth, vasculature, biochemical metabolism and molecular changes in both human and preclinical models [15], representing the main imaging tool used in the diagnosis of gliomas [17]. However, conventional MRI of gliomas has inherent limitations, such as inadequate assessment of both peritumoural oedema and integrity of the adjacent white matter [18,19]. The use of conventional paramagnetic or superparamagnetic contrast media allows the identification of areas with blood-brain barrier (BBB) disruption [20]. In addition, the recent molecular imaging approaches enable researchers to visualize molecular events associated with tumour proliferation and invasion, bringing the potential of diagnostic imaging to the cellular and molecular aspects of tumour biology [21]. Advanced magnetic resonance imaging techniques such as diffusion-weighted (DWI) and perfusion-weighted imaging (PWI) methods can provide useful information on tumour location and extent of growth, brain invasiveness, regional blood flow and blood volume, inflammation and tumour cellularity. All of these tumour features/characteristics are specific for different glioma grades and can offer an invaluable help in patient prognosis [20,22,23]. Moreover, magnetization transfer (MT) imaging is believed to provide a non-specific indicator of the structural integrity of the tissue, being different in tumours compared with healthy tissue because of the inflammatory processes that take place in the lesion [24]. Finally, magnetic resonance spectroscopy (MRS) offers the possibility of obtaining *in vivo* information about tumoural metabolism, identifying the metabolites that better correlate with neoplastic progression [25].

As far as we know, MRI and MRS have not been used to study either gender differences in brain tumourigenesis or to assess differences in the tumoural microenvironment characterization depending on the sex. The last objective of this work is to estimate whether gender differences could play a role in the biology and aggressiveness of glioblastoma multiforme. The C6 glioma rat was used as an experimental model of GBM to test differences in tumour growth and invasion [26], and tumour features like cellularity, oedema, necrosis, macromolecular environment, haemodynamic parameters and metabolism were evaluated by *in vivo* MR techniques. Metabolic behaviour was also analysed by placing rats in automated behavioural monitoring home cages [27]. Finally, postmortem BBB permeability was assessed by measuring extravasation of Evans blue-albumin [28] and glutamate and glutamine by high-performance liquid chromatography (HPLC). The putative glioblastoma biomarker glial fibrillary acidic protein (GFAP) [29] was also measured in the blood serum.

## Methods

### Animal handling

The experimental procedures were approved by the ethical committee of our centre (Instituto de Investigaciones Biomédicas ‘Alberto Sols’) and met the national (R.D. 53/2013) and the European Community guidelines (2010/62/UE) for care and management of experimental animals. The rats were housed in the animal premises of our institution (Reg. No. ES280790000188) and cared by specialized personnel.

Albino Wistar rats (*Rattus norvegicus*) were used (body weight (b.w.) 230 ± 20 g). The animals were kept in cages in a controlled room, with a 12-h cycle of light and darkness, temperature of 22°C ± 2°C and water and food access *ad libitum*. Four different groups of animals (*n* = 8 rats per group) were employed, two of them constituted tumour-bearing rats divided in two sub-groups of male and female individuals, and the other two were sham groups of male and females also. An additional cohort of 20 rats grouped in the same four experimental conditions (*n* = 5) was used to measure spontaneous energy metabolism parameters and to perform postmortem analysis by duplicate.

### Tumour implantation

Authenticated rat glioma C6 cells were obtained from the American Type Culture Collection (ATCC number CCL-107) (Manassas, VA, USA). The cells were grown in Dulbecco's modified eagle medium-4-(2-hydroxyethyl)-1-piperazineethanesulfonic acid (DMEM-HEPES) to which 10% fetal bovine serum (FBS) (Gibco®, Thermo Fisher Scientific, Inc., Waltham, MA, USA) and antibiotics were added. The animals were intraperitoneally (i.p.) anaesthetized (with a mixture of ketamine hydrochloride (75 mg/kg b.w.) and medetomidine hydrochloride (0.5 mg/kg b.w.)) and placed in a stereotaxic device where 10^5^ C6 cells in 10 μL of medium were injected in the right caudate nucleus [30]. After surgery, the rats were induced to recover with atipamezole hydrochloride (5 mg/kg b.w.) administrated subcutaneously (s.c.). They also received meloxicam s.c. as analgesia (0.5 mg/kg b.w.) during the following 3 days. Sham animals were submitted to the same procedure with an intracranial injection of 10 μL of DMEM-HEPES alone.

### Magnetic resonance experiments

*In vivo* MR experiments were performed on a Bruker Pharmascan system (Bruker Medical GmbH®, Ettlingen, Germany) using a 7.0-T horizontal superconducting magnet, equipped with a 38-mm diameter ^1^H selective birdcage resonator and a gradient insert with 90-mm diameter and a maximum intensity of 360 mT/m. All data were acquired by running the ParaVision 5.1. software (Bruker Medical Gmbh®) operating on a Linux platform. Anaesthesia was initiated in an induction box through inhalation of oxygen (1 L/min) containing 3% to 4% of isofluorane and maintained during the experiment by employing a nose mask with a flow of 1% to 1.5% of isofluorane in O_2_. The animals were placed in a heated probe, which maintained the core body temperature at approximately 37°C. The physiological status of the rats was monitored by a gating system designed for small animals (SA Instruments, Inc., Stony Brook, NY, USA) using the respiratory rate and body temperature.

#### Anatomical images

T_2_-weighted (T_2_W) spin-echo (SE) images were obtained with a rapid acquisition with relaxation enhancement (RARE) sequence and the following parameters: repetition time (TR) = 3,000 ms, echo time (TE) = 60 ms, averages (Av) = 3 and RARE factor = 8. Contrast-enhanced T_1_-weighted (CE-T_1_W) SE images after i.v. administration of 0.3 M Gd-diethylene triamine pentaacetic acid (DTPA) (from Magnevist®, Bayer, Whippany NJ, USA), at a dose of 0.3 mmol/kg b.w., were acquired using TR = 300 ms, TE = 10 ms and Av = 3. Also, the T_2_W and T_1_W images were acquired with the geometry parameters: acquisition matrix (Mtx) = 256 × 256, field of view (FOV) = 3.5 × 3.5 cm^2^ corresponding to an in-plane resolution of 136 × 136 μm^2^, slice thickness = 1.5 mm and five slices in axial orientation.

#### Magnetization transfer contrast imaging

Magnetization transfer ratio (MTR) maps were generated from two equivalent sets of images using TR = 2,500 ms, TE = 10 ms, Av = 1 and Mtx = 128 × 128 that yielded an in-plane resolution of 273 × 273 μm^2^. The imaging set acquired with an MT pulse turn on employs a radiofrequency pulse train (*N* = 50) of bandwidth = 550 Hz, length = 5 ms, power = 5.5 μT and offset = 1,500 Hz. The MT effect was calculated according to Equation 1:

1 $$ \%\;\mathrm{MTR}=\frac{S_0-{S}_{\mathrm{MT}}}{S_0}\times 100, $$

where *S*_MT_ is the signal intensity of a pixel in the image with the application of the MT pulses, and *S*_0_ is the signal of the same pixel without it.

#### Diffusion tensor imaging

Diffusion studies were performed by running a Stejskal-Tanner sequence with a single-shot echo-planar (ss-EPI) readout gradient, where TR = 3,000 ms, TE = 40 ms, Av = 1, Mtx = 128 × 128 with an in-plane resolution of 273 × 273 μm^2^, diffusion gradient separation (Δ) = 16 ms, diffusion gradient duration (δ) = 4 ms, one basal image (without diffusion gradient application) and two *b* factors of 300 and 1,400 s/mm^2^, corresponding to a diffusion gradient strength of 33% and 71%, respectively, applied in seven directions. The mean diffusivity (MD) and fractional anisotropy (FA) parameters were measured according to Equations 2 and 3, where the corresponding eigenvalues (*λ*1, *λ*2, *λ*3) were obtained by solving the tensor.

2 $$ \mathrm{MD}=\frac{\lambda 1+\lambda 2+\lambda 2}{3} $$

3 $$ \mathrm{FA}=\frac{\sqrt{{\left(\lambda 1 - \mathrm{MD}\right)}^2+{\left(\lambda 2-\mathrm{MD}\right)}^2+{\left(\lambda 3-\mathrm{MD}\right)}^2}}{2\left(\lambda {1}^2+\lambda {2}^2+\lambda {3}^2\right)} $$

#### Perfusion-weighted imaging

Cerebral perfusion acquisitions were carried out with the *bolus tracking* technique [31] using a ss-EPI sequence and a 0.3 M Gd-DTPA solution. Briefly, the contrast agent was injected in the dorsal vein of the tail as a bolus (0.3 mmol/kg b.w.) 10 s after the acquisition start point, with TR = 250 ms, TE = 7.1 ms, Av = 1, flip angle = 30° and 150 repetitions that drove a total acquisition time of 38 s. The in-plane resolution was 437 × 547 μm^2^ from a Mtx = 80 × 64. Pixel time-evolution signal intensities were fitted to the appropriate equations for computing the corresponding parametric images of cerebral blood volume (CBV), cerebral blood flow (CBF) and mean transit time (MTT) as we previously reported [32].

#### In vivo *spectroscopy*

A point-resolved spatial spectroscopy (PRESS) was employed, in combination with VAPOR water suppression protocol, to acquire the spectrum of a 27-mm^3^ voxel placed in the tumour (or analogous region in the control animals) using TR = 3,000 ms, TE = 35 ms and 128 number of scans. The spectra were processed with LCModel [33], a prior knowledge spectral fit software, and only the peak concentrations obtained with a standard deviation lower than 20% were accepted.

The animals were checked in the MRI system every 3 to 5 days after intracranial surgery to monitor the tumour development. All rats were subjected to the whole MR *in vivo* protocol described above between 16 to 20 days after intracranial injection of C6 cells (or DMEM), when the tumour volume reached approximately 75 to 100 mm^3^ (measured in CE-T_1_W images).

#### Imaging process and analysis

The five slices acquired in every case were computed with a homemade software (My Map Analyzer) written in MatLab v. 7.01 (MathWorks, Nattick, MA, USA) in which colour-based maps were generated by fitting the signal intensities to the appropriate equation in a pixel-by-pixel base. Two regions of interest (ROIs) placed in the core and in the periphery (enhanced outer ring in CE-T_1_W images) of the tumour were manually selected from parametric maps in glioma-bearing rats. In the sham animals, analogous ROIs were measured in similar cerebral areas. The obtained data were analysed with ImageJ (National Institutes of Health, Bethesda, MD, USA, http://rsbweb.nih.gov/ij/). For the different measured parameters, the data are presented in terms of percentage, either increased or decreased. The different values are based on the fact that the analysed brain region is exactly the same for both glioma rats and control rats, according to the general expression:

4 $$ \%\;\Delta\;\mathrm{Data}=\frac{{\mathrm{Data}}_{\mathrm{glioma}}-{\mathrm{Data}}_{\mathrm{sham}}\;}{{\mathrm{Data}}_{\mathrm{sham}}}\times 100 $$

In the perfusion maps, the whole tumour was analysed. Considering the low resolution of these images, a clear discrimination between the core and the periphery of the lesion was not possible.

### Metabolic activity analysis

Five animals of each experimental group were studied in a metabolic and motor analysis system (Phenomaster®, TSE Systems GmbH, Bad Homburg vor der Höhe, Germany). The rats were isolated in individual cages where the drinking and feeding behaviour and the spontaneous respiratory and motor activities were measured using high-precision sensors that store the data every 30 min. To ensure a proper acclimation to the modified environment before the measurements, all rats were placed in training cages (equipped with the drinking and feeding devices of the metabolic ones) 24 h prior to measurements [34]. After the training, the animals were moved to the system and parameters were collected for 60 h. System delivery also included evaluations of indirect calorimetry via the respiratory exchange rate (RER), which is the ratio between delivered CO_2_ and consumed O_2_, and provided information on the metabolic substrate used [27,34].

### Postmortem analyses

Once the animals were removed from the analysis system, the blood samples were collected from a tail vein (approximately 1 mL) to isolate blood serum. The rats were then infused through the right jugular vein at a rate of 3 mL/min, first with Ringer medium and FBS (18 g/L) for 3 min; then with a mixture of Ringer, FBS (18 g/L) and Evans blue (1 g/L) for another 3 min; and finally, 3 min with the same initial mixture of Ringer and FBS for rinsing. After sacrifice by decapitation, the tumour and a similar-sized region from the same brain area in the sham animals were excised. The tissues were kept frozen at −80°C until their posterior analysis.

#### Glutamate, glutamine and GFAP determination

The tissue was powdered and deproteinized in perchloric acid-ethanol mixture [35], and glutamine (Gln) and glutamate (Glu) levels were measured by high-performance liquid chromatography (HPLC) after derivatization with *o*-phthalaldehyde. GFAP was measured in serum by an ELISA kit (Chemicon, Millipore Co., Billerica, MA, USA), with the assay sensitivity being 1.5 ng/mL.

#### BBB permeability determination

Blood-brain barrier permeability was assessed by measuring extravasation of Evans blue-albumin [28]. Frozen brain tissues were weighted and homogenized in fivefold volume of 50% trichloroacetic acid solution. The supernatant was obtained by centrifugation (10 min, 10,000×*g*, at 4°C). Evans blue extravasation was quantified in the supernatants by measuring the fluorescence (excitation at 620 nm and emission at 680 nm) [36].

### Statistical analysis

MRI data were obtained from the parametric maps. All pixels were measured in the same brain region and at the same experimental condition. Values are presented as mean ± standard error of the mean (SEM), in both the absolute and the relative data. Statistical analyses were performed using GraphPad Prism 4 software (GraphPad Software, Inc., San Diego, CA, USA); the differences were determined by unpaired two-tail *t* test analysis with Welch's correction. In all cases, *P* values lower than 0.05 were considered to be statistically significant.

## Results

### MRI and MRS experiments

**Figure 1:**
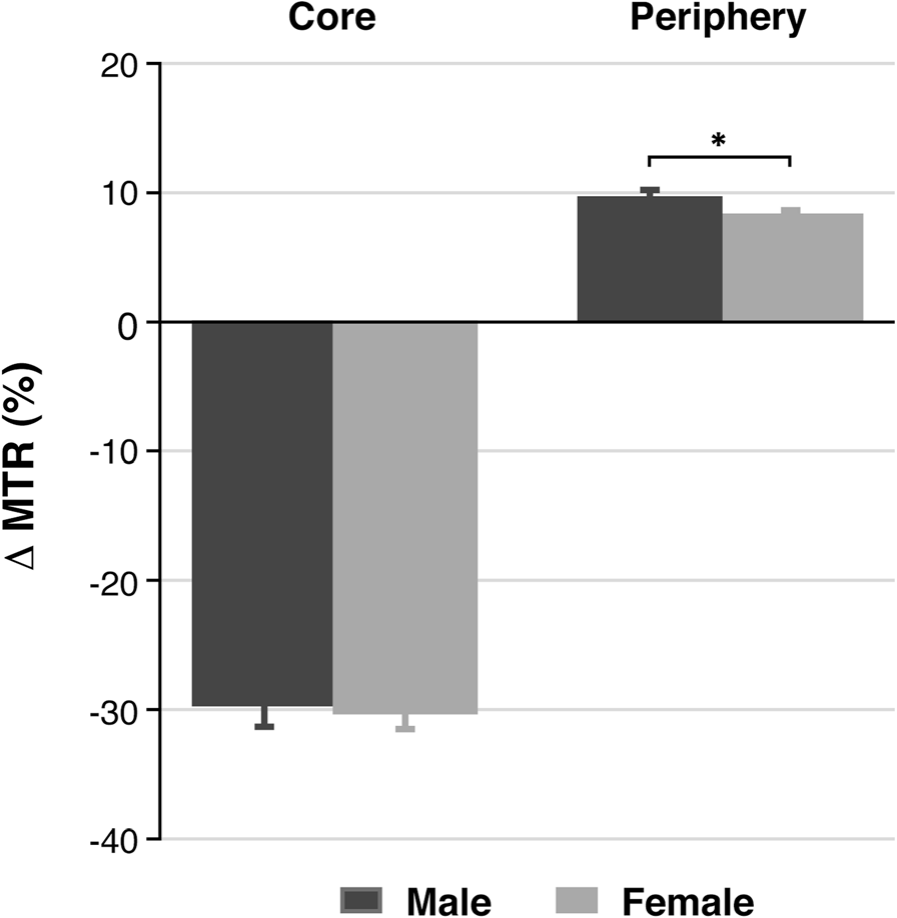
**Results from magnetization transfer in the study of gender influence in a glioblastoma rat model.** Graphic shows variation of MTR values, as a percentage, assessed in two brain regions (core and periphery of the tumour) of pathological rats related to the equivalent ones in sham animals. Unpaired *t* test analysis was performed on the data by comparing both genders in the same area. Statistically significant differences are indicated in the columns, where *P < 0.05.

Magnetization transfer studies measure the distribution of water molecules in the healthy and diseased brain, giving information about the brain macromolecular microenvironment. Results from MT studies in our experimental conditions are presented in Figure 1.

We found significant differences in the MTR values of glioma-bearing rats when compared to control animals in both regions analysed. Also, in male and female rats, there was an increase in the MTR values obtained in the periphery of the tumour, while a notable decrease was measured in the core. There was no difference between the genders regarding the MTR values in the tumoural core (−29.45% ± 0.74% for males, −29.90% ± 0.35% for females). However, a higher MTR value was seen in the periphery of tumours in males when compared to the female rats (9.44% ± 0.42% vs 8.09% ± 0.41%, *P* < 0.05).

**Figure 2:**
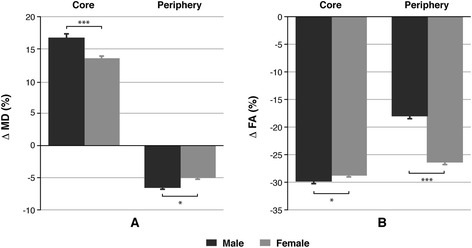
**Results from diffusion tensor in the study of gender influence in a glioblastoma rat model. (A)** Variation of MD values, as a percentage, assessed in two brain regions (core and periphery of the tumour) of pathological rats related to the equivalent ones in sham animals. **(B)** FA data in the same areas and condition. Unpaired *t* test analysis was performed on the data by comparing both genders in the same area. Statistically significant differences are indicated in the columns, where *P < 0.05 and ***P < 0.001.

Diffusion studies also provide parameters related with the structure and even the functionality of the brain [37]. Mean diffusivity measured the higher or lower water molecule restriction to the transversal motion in the tissues. Figure 2A shows the MD obtained results for both genders and two regions. Significant statistical differences were found comparing both tumoural areas, where an increase took place in the core but a decrease in the periphery. The Δ MD detected in the glioma rats in relation to the control ones was higher for males than for females both in the core (Δ MD: 16.77% ± 0.34% vs. 13.37% ± 0.22%, *P* < 0.001) and in the periphery of the tumour (Δ MD: −6.59% ± 0.22% vs. −5.03% ± 0.28%, *P* < 0.05).

**Figure 3:**
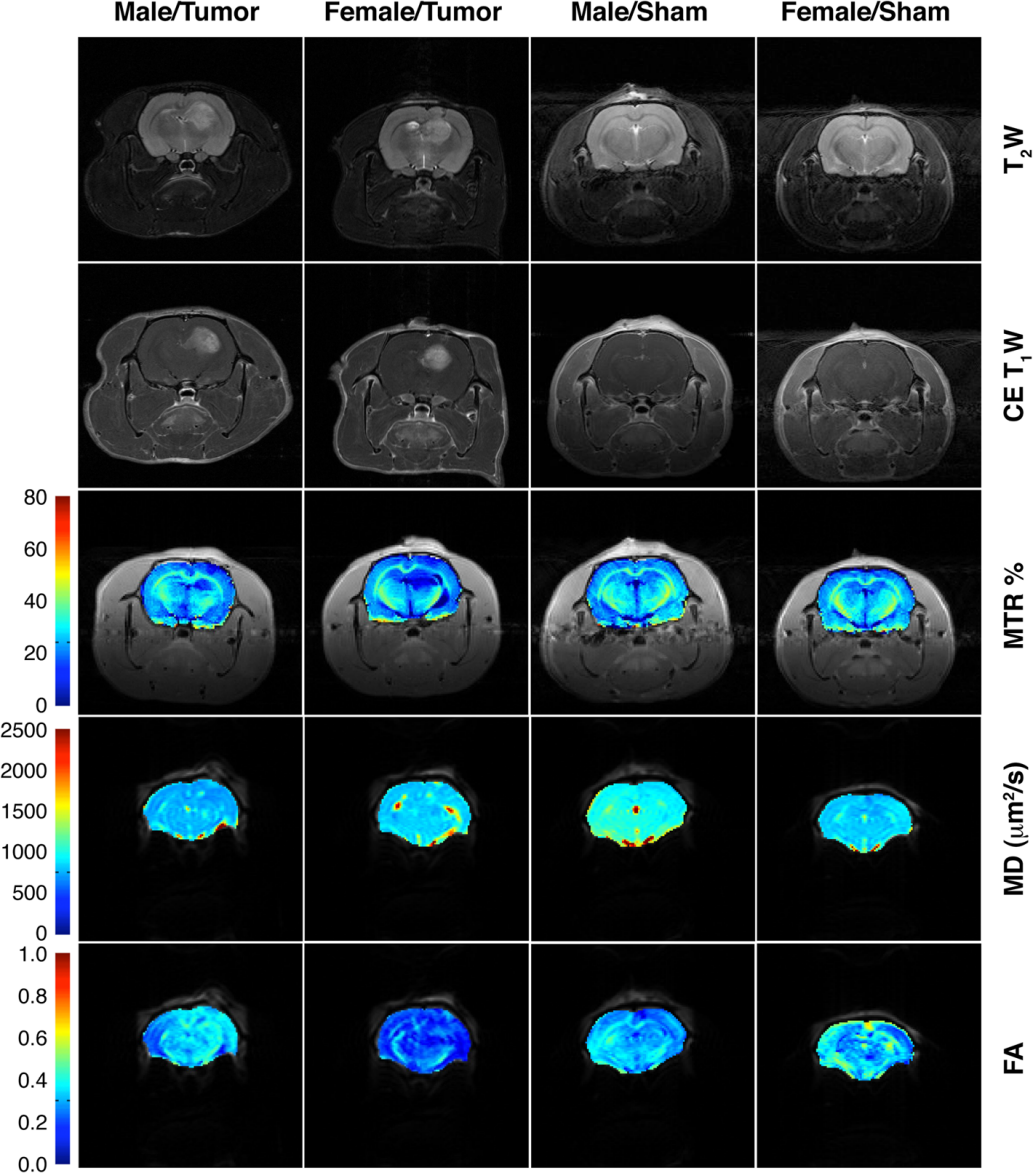
**MR images of one rat from every group in one slice.** First row images correspond to T_2_W acquisitions in which tumours can be detected as hyperintense areas. Second row shows T_1_W images, after the administration of a contrast medium that enhanced the tumoural region. Third, fourth and fifth rows present colour-base maps of MTR (%), MD (μm^2^/s) and FA measurements respectively, depicted always with dark blue those with lower values in the corresponding parameter.

In addition, fractional anisotropy maps give a value that directly correlates with the microstructure organization of the brain tissue. The data depicted in Figure 2B shows that the FA values in rats with tumour were always lower than the corresponding values of the same regions in sham animals, indicating an evident loss of integrity in the brain of these GBM model. The effect was lower for females in the core of the glioma (Δ FA: −29.78% ± 0.43% for males vs. −28.55% ± 0.39% for females, *P* < 0.05) but notably higher in the periphery (Δ FA: −18.00% ± 0.34% for males vs. −26.37% ± 0.40% for females, *P* < 0.001). In Figure 3, some representative maps of T1W, T2W, MTR, MD and FA are shown for each group of animals.

**Figure 4:**
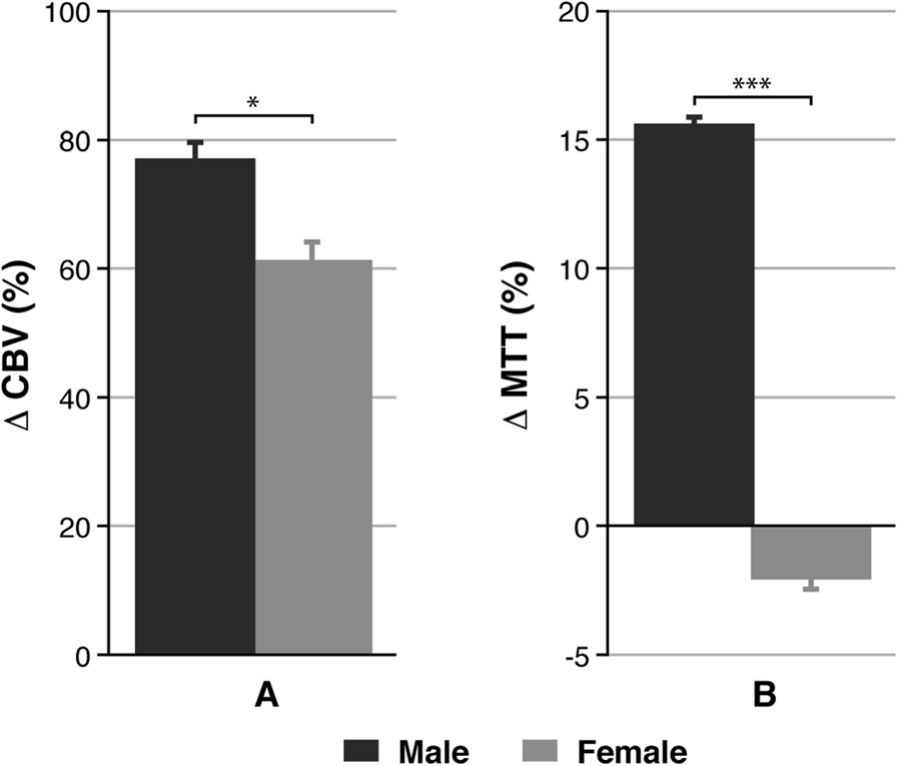
**Results obtained from perfusion in the study of gender influence in a glioblastoma rat model. (A)** Variation in the relative CBV values, as a percentage, in the tumour of pathological rats related to the equivalent ones in sham animals. **(B)** Relative MTT values in the same region and condition. Unpaired *t* test analysis was performed on the data by comparing both genders in the same area. Statistically significant differences are indicated in the columns, where *P < 0.05 and ***P < 0.001.

Besides, perfusion studies were performed to assess the cerebral integrity of the structure and function of the microvasculature which developed with angiogenic processes in the glioma models. We used the dynamic susceptibility contrast technique consistent in the acquisition of very rapid images before, during and after the administration of a bolus of Gd-DTPA to capture the microscopic magnetic susceptibility gradients generated in the first passage of the agent through the capillaries in the brain. Considering that the arterial input function was not possible to be determined in the experiment, data were normalized to an apparently healthy brain region distant from the lesion. Figure 4 shows the relative CBV (panel A) and MTT (panel B) values measured in the glioma region, when compared to an equivalent area in the control groups.

CBV values experienced a significant increase in both genders, but the difference was more accentuated in male rats (Δ CBV: 77.11% ± 2.75% vs. 61.03% ± 3.46%, *P* < 0.05). The same behaviour was detected in the regional CBF determinations (data not shown). In the case of MTT, there was an increase in males, while a decrease was measured for female rats (Δ MTT: 15.46% ± 0.35% vs. −2.06% ± 0.35%, *P* < 0.001).

The absolute MRI parameters (mean value and standard error) collected in the four experimental groups (males with tumour, females with tumour, males sham and females sham) are presented in Table 1 (tumoural core) and Table 2 (periphery of the tumour).

**Table 1:** **MRI data measured in the core of the tumour in every experimental group**

**Parameter**	**Male/tumour**	**Female/tumour**	**Male/sham**	**Female/sham**
MTR (%)	14.29 ± 0.35	13.02 ± 0.07	20.13 ± 0.23	18.57 ± 0.22
MD (μm^2^/s)	1,200 ± 9	1,120 ± 5	1,028 ± 4	988 ± 5
FA	0.244 ± 0.002	0.224 ± 0.001	0.348 ± 0.002	0.314 ± 0.002
CBV (a.u.)	1.44 ± 0.03	1.55 ± 0.04	0.81 ± 0.04	0.97 ± 0.03
CBF (a.u.)	1.56 ± 0.03	1.55 ± 0.03	0.81 ± 0.04	0.97 ± 0.03
MTT (a.u.)	1.077 ± 0.009	0.934 ± 0.006	0.933 ± 0.011	0.953 ± 0.010

Perfusion images were analysed in the whole glioma. MRI, magnetic resonance imaging; MTR, magnetization transfer ratio; MD, mean diffusivity; FA, fractional anisotropy; CBV, cerebral blood volume; CBF, cerebral blood flow; MTT, mean transit time.

**Table 2:** **MRI data measured in the periphery of the tumour in every experimental group**

**Parameter**	**Male/tumour**	**Female/tumour**	**Male/sham**	**Female/sham**
MTR (%)	22.00 ± 0.16	19.60 ± 0.12	20.10 ± 0.20	18.14 ± 0.22
MD (μm^2^/s)	963 ± 5	955 ± 4	1,036 ± 5	1,006 ± 5
FA	0.279 ± 0.002	0.232 ± 0.001	0.340 ± 0.002	0.316 ± 0.002

MRI, magnetic resonance imaging; MTR, magnetization transfer ratio; MD, mean diffusivity; FA, fractional anisotropy.

**Figure 5:**
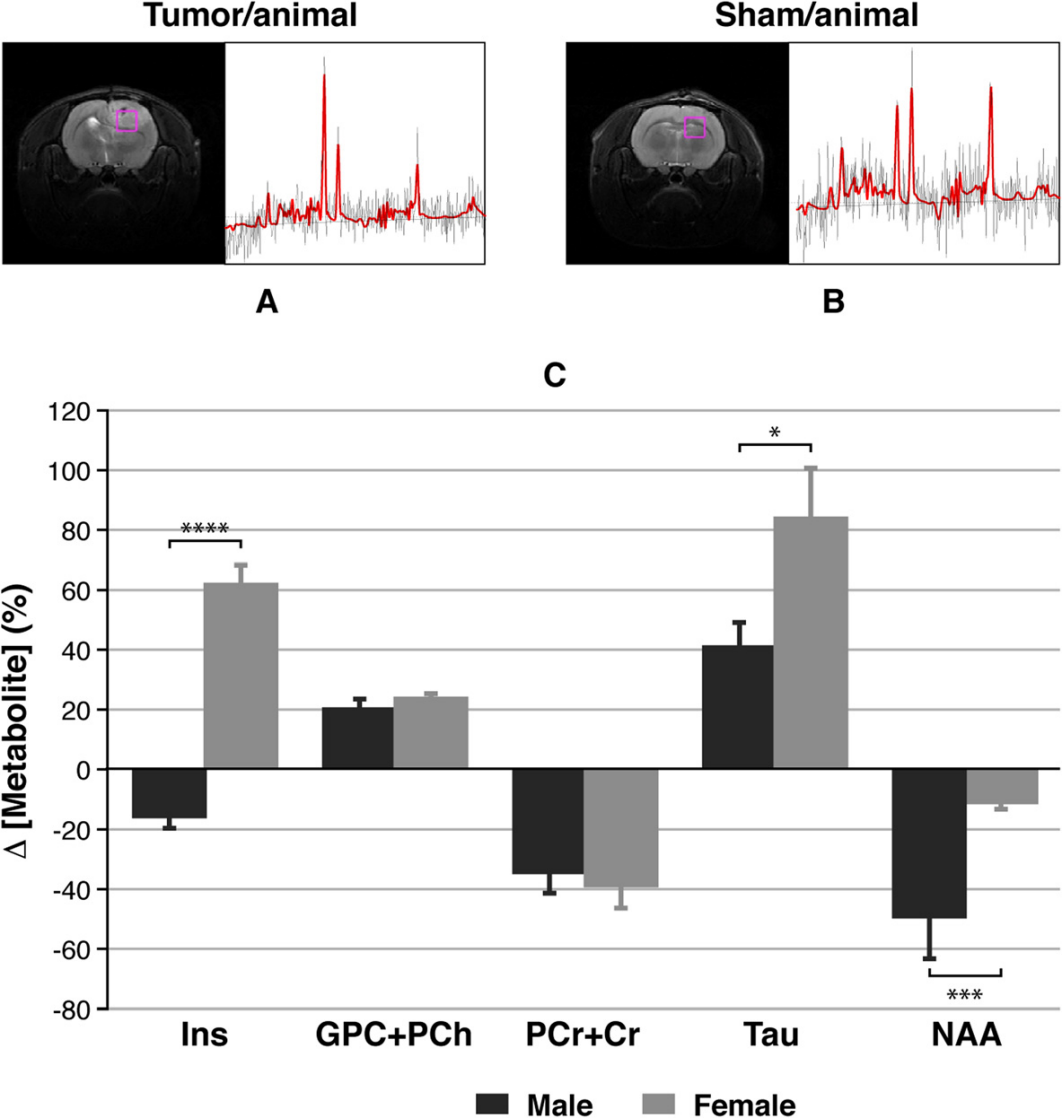
**Results from** ***in vivo*** **spectra in the study of gender influence in a glioblastoma rat model.** Upper panel depicts both examples of T_2_W images and the selected region for acquiring the spectrum in a rat with **(A)** and without **(B)** tumour and the corresponding fitted spectra. **(C)** The percentage of variation in the concentration of several metabolites detected in tumour related to sham animals in both sexes. Unpaired *t* test analysis was performed on the data by comparing both genders in the same area. Statistically significant differences are indicated in the columns, where *P < 0.05, ***P < 0.001 and ****P < 0.0001. Ins, *myo*-inositol; GPC + PCH, glycerophosphorylcholine + phosphocholine; PCr + Cr, phosphocreatine + creatine; Tau, taurine; NAA, *N*-acetylaspartic acid.

To conclude the MR analysis, we performed *in vivo* volume-selected spectra in pathological and control animals, which generated the results presented in Figure 5. The upper panels show both representative T_2_W images of a glioma-bearing rat (A) and a control rat (B) and the corresponding spectra. Panel C indicates the percentage of variation of different compounds in the tumour related to the same region in sham animals. Metabolites such as glycerophosphorylcholine + phosphocholine (GPC + PCh) presented an increase in both sexes, while phosphocreatine + creatine (PCr + Cr) showed a decrease, but neither GPC+ PCh nor PCr + Cr presented significant differences when the males were compared with females (data are presented in Table 3).

**Table 3:** **Magnetic resonance spectroscopy** ***in vivo*** **results**

	**Ins**	**CPC + PCh**	**PCr + Cr**	**Tau**	**NAA**
Male	−16.0 ± 4.2	19.8 ± 3.6	−35.3 ± 6.7	41.2 ± 8.2	−49.9 ± 14.5
Female	62.4 ± 6.2	23.8 ± 1.2	−39.5 ± 7.9	84.7 ± 16.9	−11.6 ± 2.1

The values express the percentage of variation in the metabolite concentration measured in the glioma of pathological individuals related to an analogous healthy region in brain of sham animals for every sex Ins, *myo*-inositol; GPC + PCH, glycerophosphorylcholine + phosphocholine; PCr + Cr, phosphocreatine + creatine; Tau, taurine; NAA, *N*-acetylaspartic acid.

Nevertheless, *N*-acetylaspartic acid (NAA) levels had an overall decrease, with this decay being more accentuated in males (−49.9 ± 14.5 vs. −11.6 ± 2.1, *P* < 0.001). Taurine (Tau) increased in both sexes, being the effect higher for females (41.2 ± 8.2 vs. 84.7 ± 16.9, *P* < 0.05). *Myo*-inositol (Ins even experienced a different behaviour depending on the animal gender. It suffered a notable increase in females but a decrease in male rats (62.4 ± 6.2 vs. −16.0 ± 4.2, *P* < 0.0001).

### Metabolic and motor activity

**Figure 6:**
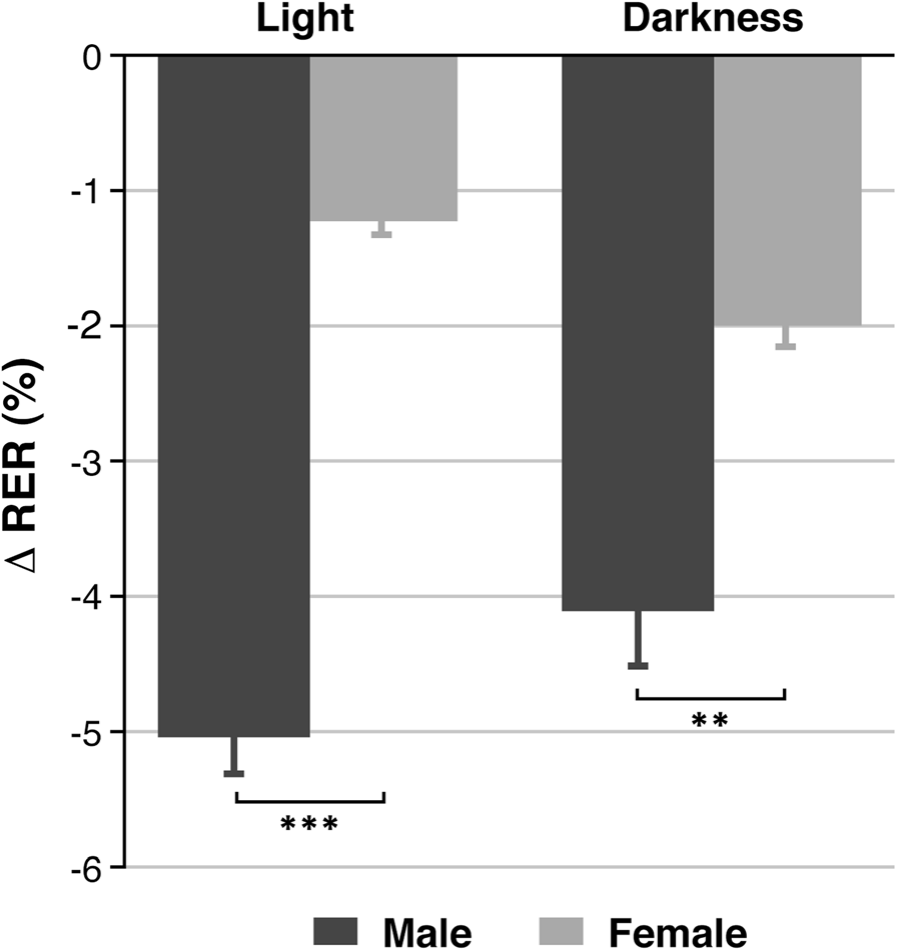
**Results of energy metabolism in the study of gender influence in glioblastoma rat model.** The graphic shows the obtained RER values. Data are divided in two circadian cycles (day and night). Unpaired *t* test analysis was performed on the data by comparing both genders in the same area. Statistically significant differences are indicated in the columns, where **P < 0.01 and ***P < 0.001.

The results obtained from motor analysis test did not yield significant differences, either between genders or between pathologic and healthy animals. The assessment of water and food intake was also similar between the experimental groups (data not shown). Nevertheless, the respiratory exchange rate, which measures the energy metabolism of the animals, declined in tumour-bearing animals when compared to the healthy controls. Once again, the RER decrease was dependent on the gender of the animal, being emphasized between day (−4.85 ± 0.24 for males vs. −1.22 ± 0.09 for females, *P* < 0.01) and night (−4.11 ± 0.21 for males vs. −1.99 ± 0.14 for females, *P* < 0.01). This difference was superior for males during the light period and for females at darkness (data shown in Figure 6).

### Postmortem analysis

**Figure 7:**
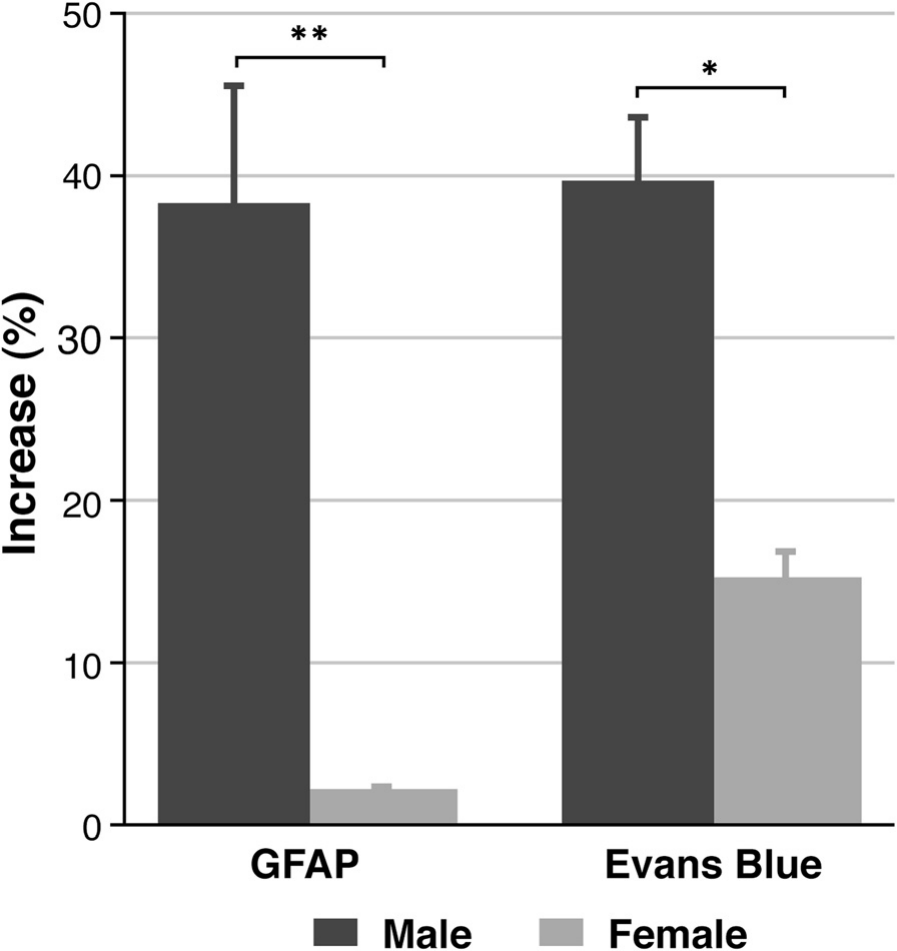
**Results from postmortem analysis of plasma and tumoural tissue in the study of gender influence.** Left bars correspond to the GFPA determination in rats measured as a percentage of the same value in sham animals. Right bars show the values in the measurement of the Evans blue accumulation. Unpaired *t* test analysis was performed on the data by comparing both genders in the same area. Statistically significant differences are indicated in the columns, where *P < 0.05 and **P < 0.01.

Finally, excised gliomas and equivalent healthy brain regions from the control rats were analysed. The peripheral glioma marker GFAP was increased in the tumoural animals (Figure 7) and significantly higher in the male group compared to the females (38.04 ± 7.22 vs. 2.25 ± 0.03, *P* < 0.01). Blood-brain barrier integrity was assessed by measuring Evans blue-albumin extravasation. In diseased animals, the BBB permeability was also larger in males than in female rats (39. 43 ± 3.94 vs. 15.19 ± 1.52, *P* < 0.05).

In order to analyse the role of glutamine and glutamate in glioma development related to the animal gender and because these two metabolites are difficult to distinguish an isolate *in vivo* with MRS, we measured the tissue concentration of Glu and Gln by HPLC in tumours and control regions. Both Gln and Glu concentration was notably increased in male glioma related to sham (15.6% ± 1.6% Gln, 8.5% ± 0.9% Glu). In contrast, both metabolites only experienced a slight increase for female animals (3.0% ± 0.2% Gln, 0.25% ± 0.01% Glu). In both cases, differences between males and females were statistically significant (*P* < 0.0001 for Gln and *P* < 0.001 for Glu).

## Discussion

In the present work, we performed a multimodal approach in order to assess whether gender influences the development of the glioblastoma by using the C6 glioma model developed in Wistar rats. We carried out an *in vivo* multiparametric analysis of the animals by using magnetic resonance imaging and spectroscopy. The determination of MR parameters linked to physiological features in the tissues [38-40] allowed us to identify important physiopathological sexual dimorphism in this model of brain cancer. We also evaluated the basal energy metabolism and neurological alterations (expressed as changes in motor activity), and finally, we made a postmortem analysis to measure the glioblastoma biomarker GFAP to assess the BBB integrity and to determine the content of Gln and Glu in the tumours.

Magnetization transfer contrast imaging is based on the contribution to the MR signal of the two different water pools present in the tissues, corresponding to the free water or mobile molecules (with a long T_2_) and the less mobile protons bounded to macromolecules and membranes (with a short T_2_) [41]. This phenomenon in a tumour is related to the cell proliferative rate, inflammation and oedematous processes, and necrosis [24,39]. As the density of cells increases, the amount of membrane macromolecules becomes higher and so the water molecules bounded to them. In our studies, the MTR in the glioma-bearing rats relative to the sham groups notably decreased in the core of the tumour presumably due to the presence of necrotic areas without any significant difference between genders. Nevertheless, the percentage of MTR was higher in the tumour periphery, suggesting that this area is the most proliferative region. This effect was sex dependent, being higher in males than in females. The results point to a higher proliferation rate in gliomas on male animals and thus an increased malignant potential of the tumoural evolution in this sex.

These assumptions were supported by the results obtained from diffusion studies. The values of MD and FA measured are also dependent on the cerebral microstructure including cellularity, extracellular tortuosity and swelling or shrinking cell processes associated with the presence of inflammation and vasogenic or cytotoxic oedema [38,42]. Many studies demonstrate that a decrease of the MD indicates that the movement of the water molecules across the extracellular matrix was hindered due to a higher cell density in the core region of the tumour compared with the surrounding tissue. The effect was major in males in accordance with magnetization transfer results that suggest a higher cell proliferation rate for this sex. However, variation of the MD values experienced an increase in the core of the tumour compared with sham animals in both sexes, suggesting the presence of necrosis and/or vasogenic oedema. This phenomenon was also notably superior in male rats than in females. A higher disruption of the BBB in glioma-bearing males was further confirmed by measuring the extravasation of Evans blue, suggesting a strong vasogenic oedema in this group. On the other hand, FA values of pathological compared with healthy animals were decreased in both regions as expected due to the loss of integrity in the brain microstructure as the tumour grows. This decrease was again higher for males in the core but unexpectedly lower in the tumour periphery. Although we have to dip in that, maybe one of two phenomena, or both, is taking place to yield this result. On one side, a better organization of this tumoural region was reported by Zhang et al. who found high diffusion directionality in the rims of rat brain gliomas demonstrating the existence of an elevated level of organization at the cellular level inside these tumours [43]. This study was performed using only males, and in view of our results, we can anticipate that this effect would have at least less significance in females in our GBM model. On the other hand, Lope-Piedrafita et al. published a longitudinal diffusion tensor imaging (DTI) analysis of C6 glioma rats showing high FA values in the brain tissue surrounding the tumour due the great glioma proliferation rate [44]. In that case, studies were carried out in females, and because the FA value in our pathological animals was higher for males, the lower decrease of FA in glioma for this gender compared to sham animals suggests a minor aggressiveness in glioma of females.

Clinical perfusion MRI measurements are currently recognized as one of the most powerful tools to assess tumour vascularization and response to treatment through the evaluation of haemodynamic parameters [20]. While CBV can be considered an indicator of the blood supplied to feed the lesion, MTT has to be taken as a marker of the microvessel density and their aberrant distribution in the tissue [40]. In this line, PW imaging gives crucial information related to angiogenic events in the glioma and viability of the vessels in the brain. In our study, glioma-bearing animals were depicted to have higher values of CBV compared with sham rats, and the increase was significantly higher in male than in female rats. This may be related to a different extension of angiogenesis depending on the gender. Additionally, MTT determinations showed a superior tortuosity and/or density of vessels inside the glioma of male animals, which together with previous parameters corroborates a higher malignancy in males.

Molecular results from *in vivo* MR spectra pointed some interesting features. The expected and reported decrease in NAA levels in glioma animals caused by the development of the C6 glioma [45] was significantly higher in male animals, in concordance with previous results that indicate a poor prognosis of the tumour in this sex. The use of total choline as a malignancy marker in clinical routine is widely extended nowadays, by using not only MRS approaches but also different imaging techniques [46]. In our studies, choline-containing compounds probably increased as a consequence of the exacerbated turnover of cell membranes due to the aggressiveness of these tumours [47], but without differences between sexes. Also, the decrease in creatine-containing compounds occurred without gender differences. In contrast, significant differences were found in the measurement of Ins and Tau, highly dependent on the animal sex. Concentration of *myo*-inositol was notably increased in females and decreased in males. It is reported that this metabolite is related to the grading of gliomas, being the lower level of Ino as an indicator of higher malignancy of the tumour [48]. In our case, we can assume a lower aggressive or malignant behaviour for female rats with glioma, in which Ins increased. Nevertheless, it is important to mention here that Ino signal usually overlaps with Gly resonance, making them hardly distinguishable from *in vivo* spectra, especially at short TE values as our case is. Gly has been reported to be increased in GBM [49], so we could not affirm that an increase in this metabolite is not contributing to the high intensity signal in female rats. But because experimental results point to a major malignancy in males, we favour to think that the increase in Gly would not be higher for females and what we are measuring in the MRS signal at approximately 3.5 ppm is predominately the Ins contribution. Testing taurine concentration, we checked that this compound level augmented in all glioma animals, but the effect was significantly superior for females. This might be due to specific functions of this amino acid, being either protective or involved in the proliferation of cells. Tau is increased in malignant gliomas in correlation to the stage of cell proliferation and the presence of oedema [50]. According to that and the MRI results, we would expect a higher Tau increase in males than in females, so we could also speculate that the presence of high taurine in the tumours could be playing a protective effect in female rats. This is in concordance with reported studies that indicate that taurine MRS signal is an apoptotic biomarker that is independent of tumour necrotic status [51]. In any case, a better understanding of the role of Tau in the development of this glioma model needs further studies. Finally, in the assessment of glutamate and glutamine, the individual contribution was not possible to isolate from ^1^H spectra, and even the total Glu + Gln peak can be contaminated with gamma-aminobutyric acid (GABA) resonances. So, we performed a *postmortem* analysis of tumour with HPLC to determine the concentration of each compound establishing that glutamine and glutamate, specially the former, were increased in glioma male groups, while there was almost no variation measured for female rats. In terms of metabolic function, Gln is strongly involved in anabolic pathways. In particular, through aspartate, Gln is essential for the synthesis of purine and pyrimidine bases as a carbon and nitrogen donor [52]. This phenomenon seems to be important for the adaptation of tumours in the transition from aerobic to anaerobic state [53]. Furthermore, it has been hypothesized that Gln serves as an energy fuel through glutaminolysis, which seems to be a prerequisite for tumour cell growth [54]. The reason why glutamine is not significantly increased in the female tumour group in our rat model is unknown and deserves future studies taking also into account that gender differences have been reported in healthy subjects in some brain areas [55]. These results can suggest the intriguing possibility that metabolism of brain tumour cells might be different depending on sex as shown in healthy subjects [56] or patients with Alzheimer' disease [57]. Besides, gender differences have been described in neuronal metabolism studied in *in vitro* model. In primary cell culture, neurons from males undergo autophagy and die faster, whereas neurons from females mobilize fatty acids, accumulate triglycerides, form lipid droplets and survive longer [58]. Sharma et al. also found that neuronal death pathways following hypoxia-ischemia are sexually dimorphic [59].

As far as we know, this is the first study on gender influence on the motor and metabolic activity in a rat model of GBM using a multichannel system with individual cages. The RER factor by circadian cycles (a linear indicator of energy metabolism activity) was decreased in tumour-bearing animals compared with the control animals, both in female and in male rats, but this decay was always higher for the latter and the difference was statistically significant both at light and at darkness cycles. A lower RER value shows a greater fat metabolism, whereas an increased RER should indicate a greater carbohydrate metabolism. Previous studies reported that during low to moderate intensity exercise, women maintain a lower RER when compared to men, being the presented results consistent with these findings of a sex difference in basal activity levels [60,61].

To end, we measured the putative peripheral GBM biomarker that is highly specific for cells with astrocytic differentiation and is widely used as a reliable marker in the immunohistochemical diagnosis and differentiation of brain [62]. In human studies, it has been shown that the expression of GFAP is increased in the serum of patients with glioblastomas compared to other groups of patients with neural tumours and healthy ones [29,63]. We found an increased expression of serum GFAP more prominent in glioma-bearing male rats. Several physiopathological mechanisms may explain increased GFAP levels in serum of GBM patients, including disruption of BBB and/or the extent of tumour angiogenesis or tumour necrosis. The fact that female tumour rats did not show a higher value of this parameter suggests that the increase in BBB permeability in the glioma of male rats, as has been exposed previously, might likely contribute to the gender difference in GFAP, although other mechanisms cannot be ruled out and needs further investigation.

## Conclusions

Brain tumourigenesis and growth assessed in C6 glioblastoma animal model is different between male and female rats. These differences encompass several MR parameters related to structural, functional and molecular features of the tumour. Most of them presented sexual dimorphism suggesting a higher malignancy and aggressiveness for male gender. In any case, the results hold substantial differences in the tumour behaviour for both genders. There were specially detected alterations in values linked to proliferation rate, inflammatory and/or oedematous processes and blood-brain barrier integrity. These MR parameters can be used as surrogate markers of the disease progression and validation of targeted therapies. Since relevant sex differences have emerged in this study, we suggest that the gender should be always considered in those studies aimed at investigating tumour behaviour (growth, angiogenesis, inflammation, proliferation rate and metabolism) and new pharmacological treatment in glioblastoma research.

## Abbreviations

BBB: blood-brain barrier
CBF: cerebral blood flow
CBV: cerebral blood volume
CE-T1W: contrast-enhanced T1-weighted
Cho: choline
Cr: creatine
DWI: diffusion-weighted imaging
FA: fractional anisotropy
FOV: field of view
GBM: glioblastoma multiforme
GFAP: glial fibrillary acidic protein
MD: mean diffusivity
MT: magnetization transfer
MTR: magnetization transfer ratio
MTT: mean transit time
NAA: *N*-acetyl aspartate
PWI: perfusion-weighted imaging
RER: respiratory exchange rate
ROI: region of interest
T1: T1-weighted
T2: T2-weighted
TE: echo time
TR: repetition time
